# Incidence and Predictors of First Line Antiretroviral Regimen Modification in Western Kenya

**DOI:** 10.1371/journal.pone.0093106

**Published:** 2014-04-02

**Authors:** Seth Inzaule, Juliana Otieno, Joan Kalyango, Lillian Nafisa, Charles Kabugo, Josephine Nalusiba, Daniel Kwaro, Clement Zeh, Charles Karamagi

**Affiliations:** 1 Kenya Medical Research Institute, Kisumu, Kenya; 2 Jaramogi Oginga Odinga teaching and Referral Hospital, Kisumu, Kenya; 3 Makerere University Medical School, Clinical Epidemiology Unit, Kampala, Uganda; 4 US Centers for Disease Control and Prevention, HIV-Research Branch, Kisumu, Kenya; 5 Division of HIV/AIDS Prevention, Centers for Disease Control and Prevention, Atlanta, Georgia, United States of America; Fundacion Huesped, Argentina

## Abstract

**Background:**

Limited antiretroviral treatment regimens in resource-limited settings require long-term sustainability of patients on the few available options. We evaluated the incidence and predictors of combined antiretroviral treatment (cART) modifications, in an outpatient cohort of 955 patients who initiated cART between January 2009 and January 2011 in western Kenya.

**Methods:**

cART modification was defined as either first time single drug substitution or switch. Incidence rates were determined by Poisson regression and risk factor analysis assessed using multivariate Cox regression modeling.

**Results:**

Over a median follow-up period of 10.7 months, 178 (18.7%) patients modified regimens (incidence rate (IR); 18.6 per 100 person years [95% CI: 16.2–21.8]). Toxicity was the most common cited reason (66.3%). In adjusted multivariate Cox piecewise regression model, WHO disease stage III/IV (aHR; 1.82, 95%CI: 1.25–2.66), stavudine (d4T) use (aHR; 2.21 95%CI: 1.49–3.30) and increase in age (aHR; 1.02, 95%CI: 1.0–1.04) were associated with increased risk of treatment modification within the first year post-cART. Zidovudine (AZT) and tenofovir (TDF) use had a reduced risk for modification (aHR; 0.60 95%CI: 0.38–0.96 and aHR; 0.51 95%CI: 0.29–0.91 respectively). Beyond one year of treatment, d4T use (aHR; 2.75, 95% CI: 1.25–6.05), baseline CD4 counts ≤350 cells/mm^3^ (aHR; 2.45, 95%CI: 1.14–5.26), increase in age (aHR; 1.05 95%CI: 1.02–1.07) and high baseline weight >60kg aHR; 2.69 95% CI: 1.58–4.59) were associated with risk of cART modification.

**Conclusions:**

Early treatment initiation at higher CD4 counts and avoiding d4T use may reduce treatment modification and subsequently improve sustainability of patients on the available limited options.

## Introduction

Access to antiretroviral therapy in resource-constrained setting has increased tremendously since the WHO, 3 by 5 strategy initiative in 2005 [1]. Currently about 6.2 million people in sub-Saharan Africa are on treatment reflecting an antiretroviral treatment (ART) coverage of about 56% [2]. While the challenge for complete coverage still holds, critical benefits have been achieved with reductions in morbidity, mortality and a general improvement in the quality of life [3], [4]. The sustainability of these gains is crucial especially in times of stagnating high HIV burden and the need to match the huge investments against HIV in resource constrained settings [5]. Current concerns towards sustainability include durability of potent and well tolerated first-line regimen, resistance issues and the availability of more potent but less expensive second and third-line regimens [6].

Drug intolerability has been cited as the main reason as to why patients either modify or discontinue regimen[7]–[14]. While this may be a global concern, the situation in affluent countries is bearable owing to the treatment options available [15]. This is in contrast to the situation in resource constrained settings where treatment regimens are limited and thus there are few options for patients experiencing drug intolerance [6]. Of the 24 FDA approved antiretroviral drugs in the six available classes, only 6 from three classes are commonly in use in resource-limited settings due to cost constraints [16]. This also limits the availability of second and third-line drugs for patients experiencing treatment failure.

The frequency of treatment modification reported in resource limited setting is fairly high and ranges from 8.3 to 78.4% for switch and 13.7–21% for discontinuation [8], [11], [14], [17]. The reported high levels of treatment modification may pose a challenge to treatment programs impacting on the overall cost of ART and limiting good patient prognosis. Due to these constraints maximizing the duration of patients on initial first-line regimen and optimizing the use of well-tolerated drugs are important.

In this study, we describe the rates, reasons and factors predictive of first-line antiretroviral treatment modification from an adult cohort, at a large HIV outpatient clinic in western Kenya.

## Methods

### Study Design, Site and Patients

We conducted a retrospective cohort study at Jaramogi Oginga Odinga teaching and referral hospital (JOOTRH): the largest referral hospital in western Kenya. The hospital is located in the southwest part of the country bordering Lake Victoria and serves an area with some of the worst health indicators in the country, including high prevalence of HIV infection (15.4%, which is greater than twice that of the national 7.1% prevalence) [18], [19]. Since 2003, the hospital provides comprehensive HIV care at no cost, as part of the national ART program through a joint effort with Columbia University (MTCT-plus program), Government of Kenya and the U.S. Centers for Disease Control (CDC).

Included in this analysis were non-pregnant adults of >15 years, who initiated first-line regimen between 1^st^ January 2009 and 31st January 2011, and had at least one follow-up visit record. During the study period, the WHO 2006 guidelines for adolescents and adults adopted by MOH-NASCOP were in use [20]. The first-line regimen consisted of the NRTI backbone zidovudine (AZT) or stavudine (d4T) or tenofovir (TDF), with lamivudine (3TC) and either Nevirapine (NVP) or Efavirenz (EFV). Patients were initiated on treatment when they either had CD4 counts of ≤200 cells/mm3 or when they had WHO stage IV disease. They would then be followed up for 2 weeks after initiating treatment, monthly if stable and six months thereafter. During the visits, clinicians would collect the patient’s demographics, clinical and pharmacological information in standardized optical character reader forms, which were then transcribed into the KEMRI/CDC HIV implementation science service program (HISS) electronic database, designed mainly for data management and program evaluation. Quality control for the stored data was done at regular intervals. At the time of registration, patients were given unique identifiers different from those in the patient support centers for concealment purposes.

### Study Outcomes and Variables Definitions

The primary outcome in this analysis was time to first combined antiretroviral treatment (cART) modification, defined as the time from treatment initiation to change of one or more antiretroviral drugs used as part of the initial first-line cART. Reasons for treatment modification were based on those documented by the clinician, usually as, toxicity, treatment failure (defined as immunological failure, according to WHO 2006 guidelines as CD4 counts decrease of 50% from the on treatment peak value, or a persistent CD4 count lower than 100 cells or fall of CD4 counts to pre-therapy baseline or clinical failure defined as new or recurrent WHO stage IV condition), non-adherence, or others. In case the documented reason was recorded as “others”, further chart review at the patient support center clinic, was done to identify the exact documented reason.

Independent variables assessed were mainly demographic and clinical in nature and included age at treatment initiation, gender, baseline CD4 counts, baseline WHO clinical stage, type of NNRTI treatment in the regimen (NVP vs EFV) and the type of NRTI backbone (AZT or TDF or d4T). Baseline parameters were assessed at cART initiation, which was also the entry point for the participants in this study.

### Statistical Analysis

Baseline patient characteristics were described using percentages for categorical data and median and inter-quartile ranges for continuous data. Incidence rates were calculated as the number of events over the person years of follow-up and the confidence intervals obtained from Poisson distribution. Drug specific incidence rates were determined as rate per persons initiating the specific drug. Kaplan-Meier analyses were used to estimate the time to first cART modification. Patients were censored at the time of event or at their last clinical follow-up visit.

Cox proportional hazards models were used to determine factors associated with cART modification. Due to violation of proportionality of hazards (PH), piecewise Cox regression models were fitted in at ≤12 months and >12 months which were time periods corresponding to the time at which the hazards were proportional. Predictor variables assessed included gender, age at treatment initiation, baseline weight, CD4 counts (obtained at closest date to treatment initiation, usually taken 6 months prior or after cART initiation), WHO stage, and the patient’s cART regimen i.e. (NVP vs. EFV), (AZT vs. d4T/TDF), (TDF vs. AZT/d4T), (d4T vs. AZT/TDF). Information on baseline CD4 was missing for 178 patients (10.6% for those with cART modification and 20.5% for those who sustained treatment). The missing CD4 data was imputed by multiple imputation using chain equations (MICE) [21]. Prediction equation included WHO staging, baseline weight, age at treatment initiation, gender, time to treatment modification, treatment modification status and first-line regimens. Before imputation, continuous variables were normalized using square root transformation for age and log-transformation for baseline weight. A total of 10 imputed data sets were generated.

Variables significant at univariate analysis (*P<0.10*) were included in the multivariate models. Estimates of hazard coefficients were derived through averaging of the 10 iterations and appropriate standard errors calculated using the Rubin’s rules [21], [22].

We also assessed factors associated with specific reasons of treatment modification grouped as toxicity and contraindication (TB treatment and other drug contraindications) for which there was sufficient data to conduct the sub-analysis. All analysis was done in Stata version11 (StataCorp, College Station, Texas).

### Ethical Review

This study was approved by the ethics review committees of Kenya Medical Research Institute and Makerere University School of Medicine and the Institutional Review Board of JOOTRH. Since this was a retrospective study of already collected anonymous data, consent waiver was sought and obtained from the above Ethics reviews committees.

## Results

### Baseline Characteristics of Study Participants

A total of 1140 participants aged 15 years and above who initiated treatment between 1^st^ January 2009 and 31^st^ January 2011 were enrolled in this study. Of these 185 had no follow-up visit and were excluded. Subsequently 955 participants who met the inclusion criteria were enrolled; of these 66.5% were female. At cART initiation, median patient age was 31 years (inter-quartile range IQR 26–38), median CD4 counts (available for 777 patients) was 257 (IQR 164–358) and median weight 60kg (IQR 53–67); 53.1% of the patients started cART at WHO stage III/IV. A majority of the patients initiated a d4T containing first-line regimen (59.7%), as well as a nevirapine-containing regimen (89.1%) (Table 1). The baseline CD4 of 309 (39.8%) participants was collected post-CART at a median period of 2.1 months (IQR 1.2–4.1).

**Table 1:** Baseline characteristics of adults initiating cART at JOOTRH between January 2009 and January 2011.

	All	Changed cART	Sustained cART	Loss to follow-up
Variable	(n = 955)	n = 178)	(n = 777)	(n = 185)
Gender – n (%)				
Male	320 (33.5)	60 (33)	260 (33)	63 (34)
Female	635 (66.5)	118 (67)	517 (67)	122 (66)
Age median (IQR)	31 (26–38)	35 (29–43)	31 (26–38)	30 (25.5–39)
Baseline body weight (kg) median (IQR)	60 (53–67)	60 (54–67)	59 (53–67)	52 (58–68)
Baseline WHO clinical stage-n (%)				
I/II	538 (56.9)	83 (46.9)	455 (58.4)	74 (43.8)
III/IV	417 (53.1)	95 (53.1)	322 (41.6)	95 (56.2)
Baseline CD4 count (cells/μl) median (IQR)	257 (164–358)	216 (120–317)	268 (175–370)	290 (189–364)
Mising-n (%)	178 (18.6)	19 (10.6)	159 (20.5)	134 (72.4)
Stavudine				
Yes	563 (59.0)	133 (74.7)	347 (44.7)	110 (59.5)
No	392 (41.0)	45 (25.3)	430 (55.3)	75 (40.5)
Zidovudine				
Yes	248 (26.0)	29 (16.2)	219 (28.2)	38 (20.5)
No	707 (74.0)	149 (83.8)	558 (71.8)	147 (79.5)
Tenofovir				
Yes	140 (14.7)	16 (9.0)	124 (16.0)	37 (20)
No	815 (85.3)	162 (91.0)	653 (84.0)	148 (80)
Nevirapine				
Yes	850 (89.0)	158 (88.8)	692 (89.5)	149 (80.5)
No	105 (11.0)	20 (11.2)	81 (10.5)	33 (17.8)

4 participants who were included in the study were on triple NRTI (ABC, NVP, EFV), while 7 (4 in the study and 3 who were lost to follow up) were on PI based regimen.

### Reasons for cART Modifications

The median follow-up time from cART initiation was 10.7 months during which a total of 178 individuals modified regimen. This represented an overall incidence rate of 18.64 per 100 person years [95% CI 16.09–21.59] over 946 person years of follow-up. The rate of modification was higher in the first year post-cART (IR; 44.08 95% CI: 36.69–52.97) compared to second (IR; 11.24 95% CI: 8.67–14.58) and the third year (IR; 3.88 95% CI: 1.85–8.12).

**Figure 1:**
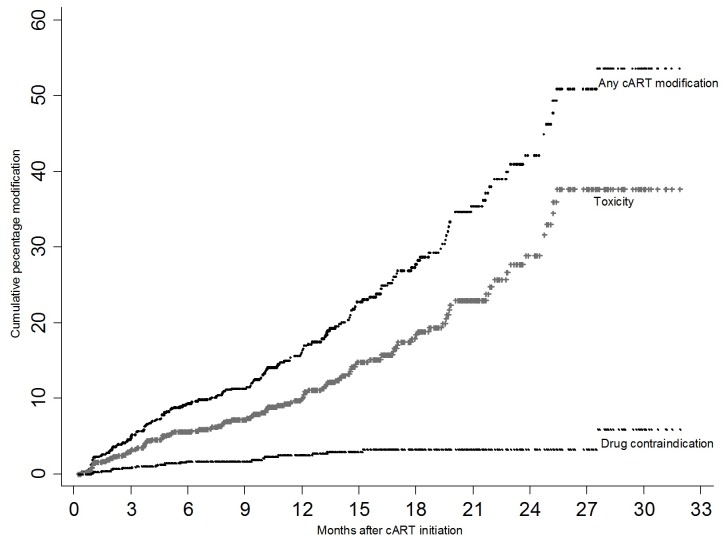
Kaplan Meier plots showing time to cART modification:overall and by key reasons of cART modifications.

Table 2 shows the reasons for cART modification as reported by the clinicians. The most commonly cited reason for modification was toxicity (66.3%, IR 12.47; 95% CI: 10.41–14.94) followed by drug contraindication (12.4%, IR; 2.33 95% CI: 1.53–3.53) while treatment failure accounted for only 2.81%, (IR; 0.53 95% CI: 0.22–1.27). A further 18.5% were recorded as either others or non-adherence (2.23%). Information on adverse events was available for 34 of 118 persons who modified regimen due to toxicity. Of these d4T related peripheral neuropathy (38.2%) and lipodystrophy (26.5%) were the most common documented drug toxicities. On the other hand modification due to contraindication was mainly of NVP to EFV substitutions (68%) as a result of rifampicin-NVP contraindication with TB patients. Figure 1 further illustrates the time to cART modification; overall and stratified by key reasons for cART modification i.e. toxicity and drug contraindications. There was a steady increase in cART modification for both overall as well as by toxicity, throughout the follow-up time. The graph for toxicity closely mimicked that for overall cART modification and this was because toxicity accounted for up to 66.3% of all modifications. On the other hand the proportion of cART modification due to drug contraindication remained steadily low at less than 5% throughout the follow-up period.

**Table 2:** Reasons of first-time antiretroviral treatment modification among 955 patients initiating first-line regimen in JOOTRH in western Kenya between January 2009 and January 2011.

Reason for cART modification	Overall	<12 months	>12 months
Toxicity -n (%)	118 (66.2)	71 (62.3)	47 (73.4)
IR (95% CI)	12.47 (10.41–14.94)	27.46 (21.76–34.6)	6.84 (5.14–9.10)
Peripheral neuropathy -n	14	5	9
Lipodystrophy -n	9	2	7
Nevirapine rash -n	7	6	1
Anaemia -n	3	3	_
Hemiparesis -n	1	1	_
Contraindications -n (%)	22 (12.4)	18 (15.8)	4 (6.3)
IR (95% CI)	2.33 (1.53–3.53)	6.96 (4.39–11.05)	0.58 (0.22–1.55)
Anti-TB drugs -n	15	11	4
Treatment failure -n (%)	5 (2.81)	2 (1.8)	3 (4.7)
IR (95% CI)	0.53 (0.22–1.27)	0.77 (0.19–3.09)	0.44 (0.14–1.35)
Others n (%)	33 (18.5)	23 (20.2)	10 (15.6)
IR (95% CI)	3.49 (2.48–4.91)	8.89 (5.91–13.34)	1.45 (0.78–2.70)
Non-adherence -n	4	3	1

A majority of cART modifications were single drug substitutions (n = 157, 88.2%), the drugs changed were d4T (n = 92), NVP (n = 48), AZT (n = 9), EFV (n = 9), TDF (n = 2). Treatment switch from first to second-line drugs accounted for 11.8% (n = 21) of all cART modifications. Overall rates for treatment modification was highest among persons initiating d4T (IR 18.83, 95% CI 15.56–22.78) based regimen as compared to either AZT (IR 4.03, 95% CI 2.17–7.49) or TDF (IR 1.43, 95% CI 0.36–5.71). This was equally the same when the rate of modifications in this NRTI’s was assessed by toxicity, treatment failure, contraindication and other reasons. Between the NNRTI’s the overall rate of cART modification was higher with EFV (IR 9.80, 95% CI 5.28–18.22) as compared to NVP (IR 7.17, 95% CI 5.58–9.21). The rate of modifications due to toxicity, treatment failure and drug contraindications was however higher with NVP as compared to EFV (Table 3).

**Table 3:** Toxicity rates of cART modification per 100 person years for individual antiretroviral regimen.

NRTI	d4T (n = 563)	AZT (n = 248)	TDF (n = 140)
Overall n (%)	108 (19.2)	10 (4.03)	2 (1.43)
IR (95% CI)	18.83 (15.56–22.78)	4.03 (2.17–7.49)	1.43 (0.36–5.71)
Toxicity n (%)	80 (14.2)	6 (2.42)	0
IR (95% CI)	13.85 (11.10–17.30)	2.42 (1.09–5.39)	_
Drug contraindication n (%)	2 (0.36)	0	0
IR (95% CI)	0.36(0.09–1.42)	_	_
Treatment failure n (%)	4 (0.71)	0	_
(IR (95% CI)	0.71 (0.27–1.89)	0.40 (0.06–2.86)	0
Others n (%)	22 (3.91)	3 (1.21)	0
(IR (95% CI)	3.91 (2.59–5.89)	1.21 (0.39–3.75)	_
**NNRTI**	**NVP (n = 850)**	**EFV (n = 102)**	**_**
Overall n (%)	61 (7.2)	10 (9.8)	
IR (95% CI)	7.17 (5.58–9.21)	9.80 (5.28–18.22)	
Toxicity n (%)	30 (3.53)	3 (2.94)	
IR (95% CI)	3.53 (2.47–5.05)	1.98 (0.50–7.92)	
Drug contraindication n (%)	19 (2.24)	1 (0.98)	
IR (95% CI)	2.23 (1.42–3.50)	0.99 (0.14–7.03)	
Treatment failure n (%)	5 (0.59)	0	
(IR (95% CI)	0.59 (0.24–1.41)	_	
Others n (%)	8 (1.42)	6 (2.42)	
(IR (95% CI)	0.94 (0.47–1.88)	5.94 (2.67–13.22)	

### Predictors of cART Modifications

Following the identification of violation of proportionality of hazard assumption for the variables baseline weight, d4T vs AZT/TDF, AZT vs. d4T/TDF and age, a piecewise Cox-regression model was fitted for two time periods ≤12 and >12 months. This time period coincided to that which the PH assumption had been met for all the variables.

In the first 12 months post cART, baseline WHO stage (III/IV vs. I/II, aHR 1.82, 95% CI 1.25–2.66), presence of d4T in regimen (aHR 2.21, 95% CI 1.49–3.30) and a yearly increase in age (aHR 1.02, 95% CI 1.0–1.04) were significantly associated with increased risk for cART modifications. On the other hand, use of either AZT or TDF was associated with reduction in risk of cART modification (aHR 0.60 95% CI: 0.38–0.96 and aHR 0.51 95% CI: 0.29–0.91 respectively) (Table 4).

**Table 4:** Predictors of cART modification.

	≤12 months		>12 months	
Variable	Crude HR	Adjusted HR	Crude HR	Adjusted HR
Gender	1.10 (0.73–1.64)		1.15 (0.69–1.90)	
Age	1.02 (1.01–1.04)	**1.02 (1.00–1.04)**	1.04 (1.02–1.07)	**1.05 (1.02–1.07)**
*Baseline CD4 (≤350 vs >350)	1.20 (0.71–1.99)	1.05 (0.63–1.75)	2.53 (1.19–5.35)	**2.45 (1.14–5.26)**
WHO clinical stage (I/II vs III/IV)	2.01 (1.38–2.92)	**1.82 (1.25–2.66)**	1.10(0.67–1.80)	1.20(0.72–2.01)
Baseline weight (≤60kg vs >60kg)	0.79 (0.55–1.15)		2.60 (1.55–4.37)	**2.69 (1.58–4.59)**
d4T vs AZT/TDF	2.40 (1.62–3.57)	**2.21(1.49–3.30)**	2.56 (1.17–5.61)	**2.75 (1.25–6.05)**
AZT vs d4T/TDF	0.52 (0.33–0.81)	**0.60 (0.38–0.96)**	0.40 (0.15–1.11)	0.43 (0.15–1.18)
TDF vs d4T/AZT	0.56 (0.31–1.00)	**0.51 (0.29–0.91)**	0.47 (0.15–1.50)	
NVP vs EFV	0.95 (0.54–1.66)		1.21 (0.52–2.81)	

Hazard ratios and 95% confidence intervals of predictors for cART modification. Bolded values indicate independent predictors. d4T-Stavudine, TDF-Tenofovir, NVP-Nevirapine, AZT-Zidovudine, EFV-Efavirenz. *178 missing CD4 values were imputed by multiple imputation using chain equations.

After 12 months post cART, a yearly increase in age (aHR 1.05 95% CI 1.02–1.07) baseline CD4 count ≤350 vs. >350 (aHR 2.45 95% CI 1.14–5.26), presence of d4T in regimen (aHR 2.75 95% CI 1.25–6.05) and baseline weight (>60 kg vs. ≤60 kg) (aHR 2.69 95% CI 1.58–4.59) were significantly associated with an increased hazard for cART modification (Table 4).

Table 5 describes the factors associated with cART modification due to drug toxicity and contraindications. Patients, who initiated a d4T containing regimen and those who were older, were significant more likely to modify regimen due to toxicity within the first year of treatment (aHR 1.93, 95% CI: 1.18–3.15 and aHR 1.03, 95% CI 1.01–1.05 respectively). After the first year of cART initiation, patients who started treatment with low CD4 counts of ≤350 vs. >350, (aHR 2.75, 95% CI: 1.05–7.21), a high baseline weight (>60 kg vs. ≤60 kg) (aHR 3.89, 95% CI 2.01–7.54), those with d4T in their regimen (aHR 3.84, 95% CI 1.37–10.75) and those who were older (aHR 1.06, 95% CI: 1.05–1.09) were more likely to modify regimen due to toxicity.

**Table 5:** Predictors of cART modification for specific reasons.

	≤12 months		>12 months	
Variable	Crude HR	Adjusted HR	Crude HR	Adjusted HR
**Drug related toxicity**				
Age	1.04 (1.01–1.06)	**1.04 (1.01–1.06)**	1.05 (1.03–1.08)	**1.06 (1.03–1.09)**
*Baseline CD4 (≤350 vs >350)	0.89 (0.48–1.66)	0.73 (0.38–1.42)	2.36 (0.98–5.70)	2.35 (0.94–5.87)
WHO clinical stage (I/II vs III/IV)	0.83 (0.28–1.38)	1.26 (0.75–2.12)	1.18 (0.65–2.12)	1.39 (0.76–2.57)
Baseline weight (≤60 kg vs >60 kg)			3.68 (1.89–7.16)	**4.14 (2.08–8.24)**
d4Tvs AZT/TDF	2.29 (1.33–3.96)	**2.23(1.28–3.88)**	4.32(1.34–13.95)	**4.85 (1.50–15.74)**
**Contraindication**				
*Baseline CD4 (≤200 vs >200)	8.23 (2.48–27.31)	**5.98 (1.78–20.14)**	0.59 (0.06–5.74)	0.58 (0.06–5.68)
WHO clinical stage (I/II vs III/IV)	7.54 (2.18–26.05)	**5.92 (1.70–20.57)**		
d4T vs AZT/TDF	6.40 (1.85–22.22)	**4.10 (1.17–14.41)**		
AZT vs d4T/TDF	0.22 (0.05–0.96)	0.37 (0.08–1.62)		
TDF vs d4T/AZT			9.95 (1.40–70.75)	10.08 (1.41–72.15)

Hazard ratios and 95% confidence intervals of predictors for cART modification due to drug related toxicities and contraindication. Bolded values indicate independent predictors. d4T-Stavudine, TDF-Tenofovir, NVP-Nevirapine, *178 missing CD4 values were imputed by multiple imputation using chain equations.

Similarly patients who initiated treatment at low CD4 counts of ≤200 vs. >200 (aHR 5.98, 95% CI: 1.78–20.14), those who had WHO clinical stage III/IV vs I/II (aHR 5.92, 1.70–20.57) those who had a d4T containing regimen (aHR 4.10, 95% CI: 1.17–14.41) were more likely to modify treatment due to drug contraindications within the first year after cART initiation. Beyond the first year of cART only patients initiating a TDF containing regimen were more likely to modify treatment due to drug contraindication (aHR 10.08, 95% CI 1.41–72.15).

### Loss to Follow-up and Missing CD4 Values

Of the 1140 participants who initiated treatment during the study period, 185 (16%) did not have any follow-up visit and thus they were probably lost to follow-up (ltfu) and were excluded in this analysis. Baseline characteristics of the participants lost to follow-up and excluded were similar to those who enrolled apart from disease stage at cART initiation, with those ltfu having advanced disease stage (p = 0.003) and were also likely to have missing CD4 counts (p = 0.001) (Table 1).

A further 178 (18.6%) participants had missing data on CD4 counts. These participants had similar characteristics to those whose baseline CD4 counts was available and differed only in WHO stage III/IV (57.9% vs. 40.4% *p<0.001*). Subsequently imputation was done for the missing CD4 values. There was no difference in the determination of predictors of cART modification when the analysis was done without the imputation, with only slight adjustments in the hazard ratios (i.e. at time periods >12 months, age (aHR 1.04 95% CI 1.02–1.07), CD4 counts ≤350 vs. >350 (aHR 2.64, 95% CI: 1.25–5.59), d4T (aHR 2.52 95% CI 1.14–5.55 and baseline weight (>60 kg vs. ≤60 kg) (aHR 2.23 95% CI 1.31–3.79). However there were differences in the predictors of cART modification due to toxicity and contraindications when analysis was done without imputation of missing CD4 counts. Low CD4 counts ≤350 vs. >350 (aHR 2.87 95% CI 1.11–7.42) was a significant predictor of cART modification due to toxicity at >12 months, in addition to age, d4T and high baseline weight. On the other hand, low CD4 counts ≤200 vs. >200 was no longer associated with cART modifications due to contraindication at ≤12 months post treatment initiation.

## Discussion

We observed a moderate incidence of treatment modification; 18.64 per 100 person years within a median follow-up period of 10.7 months in this adult cohort of patients who started cART as part of routine clinical care in a resource limited setting.

The relatively moderate rates of cART modifications are synonymous with those reported from similar settings [11], [14], [23], but are slightly higher than those observed in programs and clinical trials [14], [24]. This difference is likely due to close treatment monitoring or potential selection bias of persons enrolled in clinical trials and programs as compared to those in routine clinical settings. The rates are however still lower than those observed in developed nations where cART modifications are as high as >50% [9], [25], [26]. The difference may probably be due to limited cART options or the pre-determined population based ART guidelines in these settings, which is likely to influence the clinicians’ decision on cART modification.

Toxicity was the most common reason for cART modification similar to what has been reported in other studies [8], [10]–[14], [27]. Stavudine accounted for majority of toxicity related cART modification with risk increasing with time on treatment. Previous studies have shown a high toxicity profile for d4T-based regimen presenting mainly as acute lactic acidosis and long term mitochondrial toxicities (lipoastrophy and peripheral neuropathy)[28]–[32]. This has consequently led to the current WHO guidelines recommending d4T phase-out and the subsequent adoption of TDF or AZT drugs which have better tolerability [6]. While d4T use in affluent nations has subsequently declined, African countries still rely on d4T based regimen due to high cost of TDF [33], [34]. However cost-effectiveness analysis comparing high cost TDF to d4T showed a general preference for TDF but with relatively high cost of approximately 17 US$ per QALY increase per month [33]. In this study the presence of TDF in the first-line regimen was observed to have a 49% reduction in the risk of cART modification; an indication of its good safety profile as has been reported in other studies. Contrary however to findings from some studies was the absence of AZT risk for cART modification. In our study, patients on AZT had a 40% reduced risk, which may either imply that AZT equally had a good toxicity profile in this population or may portray the resistance by clinicians for AZT-based modifications, which may appear milder than those for d4T. Apart from the NRTI drugs, both EFV and NVP had a moderate rate of toxicity related modifications with higher rates in the first year post-cART. This is in concordance with the reported occurrence of Nevirapine (rash and hepatotoxicity) and EFV (central nervous toxicity) adverse events usually occurring at early stages of cART initiation [6].

Modifications due to drug contraindications were also significant with changes due to TB treatment accounting for the majority. This reflects the high level of TB burden in this region and the need for focused TB prevention and screening programs among HIV patients on care and treatment. Both d4T and TDF were significantly associated with risk of cART modification due to drug contraindication. This may probably be due to the reported increased risk for peripheral neuropathy when both Isoniazid TB drugs are used together with d4T [35]. On the other hand, TDF association with cART modification experienced only after one year post-cART, could have been confounded by the relatively few drug contraindication related modifications experienced after one year of treatment. However some studies have also reported potential risk of increased nephrotoxicity when TDF is used with some TB drugs (rifampicin, streptomycin and pyrazinamide) and this may probably influence TDF modification in TB patients [36].

Treatment switches due to ART failure were low at less than 1% in the studied population, which may suggest a high efficacy of first-line drugs in this region or a shorter follow-up period or the lack of proper mechanisms to identify treatment failure in such settings. Due to lack of adequate viral load and drug resistance capacity in resource limited settings, CD4 values and clinical assessment are usually used to assess treatment failure. However previous studies have shown a poor correlation of CD4 and clinical assessment with treatment failure, leading to late detection of treatment failure and subsequent late switches [37], [38]. Following this, the revised WHO guidelines now recommend the use of routine viral load as a better monitoring strategy in determining treatment response [39].

However about 2.2% of the study participants were on second-line regimen at the end of the study. This could imply that although toxicity may have been the main reason for treatment modification, it is likely that this may have been accompanied by treatment failure necessitating switch of regimen rather than single drug substitutions. This further corroborates existing evidence for toxicity mediated treatment failure through non-adherence and further calls for close monitoring of patients on treatment to prevent loss of salvageable regimen through avoidable switches.

Increase in age at cART initiation was found to have a moderate risk for modification similar to what has been observed in other studies. Baseline weight was also a significant risk factor for treatment modification, in which patients weighing over 60 kg were twice at risk for modification. This is synonymous to what has been observed in other studies showing the association between NVP and d4T based toxicities and higher baseline body weight [40], [41]. This could also explain the observed greater than four times risk of treatment modification due to toxicity for heavier persons after 12 months of treatment.

The risk of cART changes also increased with the stage of the disease as reflected in both CD4 counts and WHO disease staging. These findings are synonymous with what has been previously reported showing that sicker patients are more likely to modify regimen due to a higher risk of adverse events [42]–[44]. In addition sicker patients are also likely to be on other medications for opportunistic infections and may equally be at risk of changing treatment due to drug contraindications. These findings further build up on the evidence that treatment initiation at higher CD4 counts and at lower WHO disease stage leads to increased patient’s durability on the initial first-line regimen. In Kenya, the level of HIV status awareness is still low and majority of patients are likely to know their status mainly when they are at advanced stages of the disease [45]. This may result in poor treatment outcomes as well as increased risk of cART modifications. The current push for more aggressive HIV testing programs like provider initiated counseling and testing (PITC) and home based care and testing (HBCT) in addition to the routine voluntary counseling and testing (VCT) are likely to improve this situation, by timely placement of patients on treatment and this could subsequently reduce the risk for cART modifications.

Our study has limitations. First, being a retrospective analysis of records, various errors experienced with such a study design are likely to be present. This includes the potential for random misclassification error during clinician recording. In addition, non-specific clinician’s recording of the reasons for cART modification in some patients was non-informative as it was only recorded as “others”. There was also the potential for selection bias as about 16% of the patients were lost to follow-up. It is likely that the reasons leading to the loss to follow up may have been linked to the outcome of the study in that some of this patients may have opted out on treatment due to adverse events experienced, and this could have the potential of under-estimating the magnitude of cART modification. Moreover baseline CD4 values for some participants were collected within four months after treatment initiation. Although this may reflect delays in results relay, it could also bias the results, if the CD4 were actually determined after treatment initiation, since some patients are likely to respond quite well after treatment leading to significant difference in the baseline and 4 months post-cART CD4. Finally the study findings are limited to settings where similar regimens are in use, as in our study majority of the patients were on NVP and d4T based regimen.

Notwithstanding the limitations, this study provides unique findings with regard to incidence and predictors of cART modifications and had several strengths. First, this study was carried out in a routine clinical set-up, whose characteristics may represent the routine standard of care in most resource limited settings and thus allowing generalizability. Secondly, our study assessed the rate of cART modification at two different time periods; during the first year and after the first year post-cART initiation and provided information on associated factors for treatment modification at the two time periods as well as for major specific reasons of cART modification. This is vital in informing clinicians on the time at which patients are at risk of modifying treatment and the possible factors that could influence modification at those time periods.

In conclusion, we report a moderate rate of cART modification from a routine clinical set-up in western Kenya. Toxicity was identified as the most common reason for cART modification while factors predictive of the change were advanced WHO staging, low CD4 counts, a yearly increase in age, a higher baseline weight and the presence of d4T in regimen. On the other hand, the presence of zidovudine and tenofovir in regimen led to a reduction in the hazard for modifications.

The findings of this study have several implications for the management of patients on treatment. First, the identification of toxicity as the main reason for cART modifications calls for the need for early and proactive management of toxicity in order to prevent poor treatment outcomes including treatment failure. Second, the identification of low CD4 and advanced disease stages as important predictors for cART changes indicates that adoption of revised early treatment initiation strategies is likely to be beneficial in the prevention of cART modification. Finally, the continuous identification of d4T as an important predictor of cART modification calls for an accelerated implementation of the WHO guidelines recommending d4T phase-off in favor of TDF/AZT based regimen in resource limited settings as these is likely to significantly minimize treatment modifications.
